# Genome sequence of *Bifidobacterium canis* SY61, a sialidase-producing bacterium isolated from canine feces

**DOI:** 10.1128/mra.01142-25

**Published:** 2026-01-29

**Authors:** Yuji Yamamoto, Mayu Takahashi, Aina Yamaguchi, Nanoha Sakakura, Keita Nishiyama, Takao Mukai

**Affiliations:** 1 Laboratory of Cellular Microbiology, School of Veterinary Medicine, Kitasato University, Aomori, Japan; 2 Laboratory of Animal Food Function, Graduate School of Agricultural Science, Tohoku University 13101, Sendai, Japan; University of Wisconsin-Madison, Madison, Wisconsin, USA

**Keywords:** canine, gut microbiota, sialidase, bifidobacteria, carbohydrate metabolism

## Abstract

Sialidase facilitates the utilization of host mucin glycans and milk oligosaccharides by intestinal bacteria. We identified *Bifidobacterium canis* SY61 as a sialidase-producing canine gut bacterium and sequenced its genome, providing the first gap-free genomic resource for this species and advancing our understanding of carbohydrate metabolism in the canine intestinal microbiota.

## ANNOUNCEMENT

Sialidase, a key enzyme in intestinal carbohydrate metabolism by microbiota, catalyzes the removal of terminal sialic acids from glycans. In the gut, mucin *O*-glycans in the intestinal mucus layer are frequently modified with sialic acids, which must be cleaved to enable further degradation. Many milk oligosaccharides in humans and animals are also sialylated, requiring sialidase for their utilization (1). Although sialidase has been reported in several gut bacterial genera, including *Bacteroides*, *Clostridium*, *Bifidobacterium*, and *Akkermansia*, its distribution in the canine gut microbiota remains poorly characterized. Here, we identified *Bifidobacterium canis* as a sialidase-producing bacterium in dogs and present its gap-free genome sequence.

Fresh feces collected from 7- to 9-year-old beagle dogs raised in the kennel of the School of Veterinary Medicine, Kitasato University, were immediately suspended in saline; streaked onto Gifu anaerobic medium (GAM; Nissui Pharmaceuticals, Tokyo, Japan), Man-Rogosa-Sharp medium (MRS; Becton, Dickinson and Company, Franklin Lakes, NJ, USA), and TOS propionate medium (Yakult Pharmaceutical Industry, Tokyo, Japan) agar plates; and incubated at 37°C for 48 h under anaerobic conditions. Colonies growing on each agar plate were seeded onto GAM and incubated under anaerobic conditions for 24 h. Sialidase activity was measured from this culture using 4-methylumbelliferyl-N-acetyl-α-D-neuraminic acid sodium salt (Sigma-Aldrich, St. Louis, MO, USA), a fluorescent substrate for sialidase, as described previously (2). Subsequent evaluation of the 65 isolated strains revealed that only strain SY61, isolated from a 9-year-old female dog, exhibited activity.

To obtain genomic DNA, a single colony of SY61 was incubated overnight in half-strength MRS medium. The culture was then inoculated into the same fresh medium at a 2% concentration and cultivated until the log phase. Genomic DNA was purified using the Qiagen Genomic-tip 20/G kit (Qiagen, Hilden, Germany), according to the manufacturer’s instructions, except that mutanolysin (125 U/mL; Sigma-Aldrich) treatment was performed concurrently with lysozyme treatment. Purified genomic DNA was sheared to 10–20 kbp using a gTUBE (Covaris, Woburn, MA, USA), and libraries were prepared using the SMRTbell Express Template Prep Kit 2.0 (PacBio, Menlo Park, CA, USA), following the manufacturer’s instructions. The polymerase–library complex prepared using the Revio Binding Kit (PacBio) was sequenced on a Revio platform. Sequencing data were processed using SMRT Link v12.0.0.177059 (PacBio) to remove overhang adapter sequences and generate circular consensus sequencing (CCS) reads. CCS reads longer than 1,000 bases were assembled using Flye v2.9.2-b1786 with default parameters. DFAST v1.2.0 (https://dfast.ddbj.nig.ac.jp/) (3) was used for completeness assessment, taxonomic assignment, and annotation of the assembled genome.

Genome analysis of the SY61 strain revealed a single circular chromosome of 2,181,470 bp, predicted to encode 1,701 CDSs. Taxonomic analysis based on average nucleotide identity (ANI) identified the isolate as *B. canis* (Table 1). GSD1FS is currently the only registered *B. canis* genome (4), making SY61 the second reported *B. canis* genome and the first gap-free genome for this species.

**TABLE 1:** Assembly statistics and general genome information

Sequence and genome information	Data for *B. canis* SY61
PacBio Revio sequencing	
No. of reads	20382
Total no. of bases	247860353
Average read length (bp)	12160.7
Coverage (×)	113.621
Chromosome features^*a*^	
Total sequence length (bp)	2181470
No. of sequences:	1
Longest sequences (bp)	2181470
Gap ratio (%)	0
GC content (%)	57.3
No. of CDSs^*b*^	1701
Average protein length (aa)	366.4
Coding proportion (%)	85.7
No. of rRNAs	12
No. of tRNAs	53
No. of CRISPR regions	4
Completeness check (%)	99.92
Taxonomy check (%) (ANI to *Bifodobacterium canis* GSD1FS [GCA_009727065.1])	99.92

^*a*^ All genomic statistics are output from the DFAST pipeline.

^*b*^ CDSs, coding sequences.

## Data Availability

This genome sequence has been deposited in DDBJ/ENA/GenBank under the accession number AP038862. Raw data is available in the DDBJ Sequence Read Archive under the accession number DRA019866.
